# DRAM1 Protects Neuroblastoma Cells from Oxygen-Glucose Deprivation/Reperfusion-Induced Injury via Autophagy

**DOI:** 10.3390/ijms151019253

**Published:** 2014-10-23

**Authors:** Mengqiang Yu, Yugang Jiang, Qingliang Feng, Yi’an Ouyang, Jie Gan

**Affiliations:** Department of Neurosurgery, Second Xiangya Hospital of Central South University, Changsha 410011, China; E-Mails: yumengqiang@live.cn (M.Y.); fengqingliang028@163.com (Q.F.); ouyangyian028@163.com (Y.O.); ganjie028@163.com (J.G.)

**Keywords:** DNA damage-regulated autophagy modulator protein 1 (DRAM1), oxygen-glucose deprivation and reperfusion (OGD/R), cerebral ischemia and reperfusion (I/R) injury, autophagy

## Abstract

DNA damage-regulated autophagy modulator protein 1 (DRAM1), a multi-pass membrane lysosomal protein, is reportedly a tumor protein p53 (*TP53*) target gene involved in autophagy. During cerebral ischemia/reperfusion (I/R) injury, DRAM1 protein expression is increased, and autophagy is activated. However, the functional significance of DRAM1 and the relationship between DRAM1 and autophagy in brain I/R remains uncertain. The aim of this study is to investigate whether DRAM1 mediates autophagy activation in cerebral I/R injury and to explore its possible effects and mechanisms. We adopt the oxygen-glucose deprivation and reperfusion (OGD/R) Neuro-2a cell model to mimic cerebral I/R conditions *in vitro*, and RNA interference is used to knock down DRAM1 expression in this model. Cell viability assay is performed using the LIVE/DEAD viability/cytotoxicity kit. Cell phenotypic changes are analyzed through Western blot assays. Autophagy flux is monitored through the tandem red fluorescent protein–Green fluorescent protein–microtubule associated protein 1 light chain 3 (RFP–GFP–LC3) construct. The expression levels of DRAM1 and microtubule associated protein 1 light chain 3II/I (LC3II/I) are strongly up-regulated in Neuro-2a cells after OGD/R treatment and peaked at the 12 h reperfusion time point. The autophagy-specific inhibitor 3-Methyladenine (3-MA) inhibits the expression of DRAM1 and LC3II/I and exacerbates OGD/R-induced cell injury. Furthermore, DRAM1 knockdown aggravates OGD/R-induced cell injury and significantly blocks autophagy through decreasing autophagosome-lysosome fusion. In conclusion, our data demonstrate that DRAM1 knockdown in Neuro-2a cells inhibits autophagy by blocking autophagosome-lysosome fusion and exacerbated OGD/R-induced cell injury. Thus, DRAM1 might constitute a new therapeutic target for I/R diseases.

## 1. Introduction

Cerebral homeostasis and function rely on an adequate supply of oxygenated blood. Stroke, often caused by hypoxic ischemic encephalopathy and acute cerebrovascular incidents, interrupts cerebral blood supply and results in high mortality and long-term disability worldwide [1]. The goal of stroke treatment is to restore the blood supply as rapidly as possible; however, reperfusion may aggravate cerebral injury. The prevention and treatment of cerebral ischemia/reperfusion (I/R)-induced injury have been considered pivotal strategies for stroke intervention. Multiple pathophysiology processes may be involved in cerebral I/R injury, including neutrophil infiltration, post-ischemic hyperperfusion, and impairment of the blood-brain barrier [2]. However, the mechanisms involved in these processes are complex and remain uncertain.

Autophagy is a basic catabolic mechanism involving intracellular components and damaged organelle degradation that maintains normal organelle function and nutrient restoration [3], and it is an evolutionarily conserved mechanism from yeast to Homo sapiens [4]. Generally, autophagy is activated by many physiological and pathological conditions. A double-membrane-bound autophagosome forms and travels through the cytoplasm of the cell to the lysosome, and the two organelles fuse into an autophagolysosome and degrade in the lysosome. The degradation products can be reused by the cell for metabolism [5]. During cerebral I/R injury, autophagy is activated, and the regulation of autophagy affects the results of the brain I/R injury. However, its effects on cerebral I/R are controversial. On the one hand, some investigators [6,7,8,9] have shown that cerebral ischemia induced autophagy-like cell death, and inhibition of autophagy prevents neuron death after ischemic injury. On the other hand, other investigators [10,11,12,13,14] have shown that autophagy activation is involved in neuroprotection in brain I/R injury. Autophagy has been considered a double-edged sword with pro-survival or pro-death potential in cerebral I/R injury [15].

DNA damage-regulated autophagy modulator protein 1 (DRAM1) is a multi-pass membrane lysosomal protein that has shown a high degree of conservation throughout evolution and has been reported as a tumor protein p53 (*TP53*) target gene involved in autophagy and apoptosis [16]. During cerebral I/R injury, DRAM1 expression is altered along with the ratio of microtubule associated protein 1 light chain 3II/I (LC3II/I) [17]; however, whether DRAM1 regulates autophagy during brain I/R is uncertain. Given that DRAM1 is mainly located in the lysosomal membrane and that lysosomes play pivotal roles in the autophagic pathway, we hypothesized that DRAM1 may play a role in autophagy through the lysosomes during cerebral I/R injury. To investigate this hypothesis, the oxygen glucose deprivation and reperfusion (OGD/R) model was used to mimic ischemia/reperfusion conditions *in vitro*. We found that OGD/R induced DRAM1-dependent stimulation of autophagy in Neuro-2a cells. Knockdown of DRAM1 inhibited autophagy flux by decreasing autophagosome-lysosome fusion.

## 2. Results and Discussion

### 2.1. Oxygen Glucose Deprivation and Reperfusion (OGD/R) Treatment Increases DNA Damage-Regulated Autophagy Modulator Protein 1 (DRAM1) Expression and Induces Autophagy Activation

**Figure 1:**
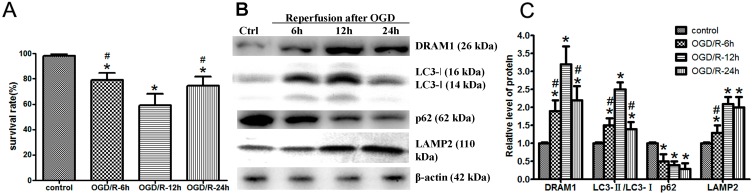
Oxygen glucose deprivation and reperfusion (OGD/R) treatment increases DNA damage-regulated autophagy modulator protein 1 (DRAM1) expression and induces autophagy activation. Neuro-2a cells were subjected to 4 h OGD and harvested after 6, 12, and 24 h. (**A**) Cell viability was assessed using the LIVE/DEAD viability/cytotoxicity kit. Neuro-2a cells were exposed to OGD for 4 h followed by reperfusion for the indicated times; (**B**) Relative protein expression levels. At the indicated reperfusion time points, Neuro-2a cells were collected, and protein was extracted to assess DRAM1, microtubule associated protein 1 light chain 3 (LC3), lysosomal-associated membrane protein 2 (LAMP2), and ubiquitin-binding protein (p62) expression levels through Western blot analysis. β-Actin was used as an internal control; and (**C**) The quantitative results from (**B**). β-Actin was used as an internal control (* *p* < 0.05 *vs.* control, # *p* < 0.05 *vs.* ODG/R 12 h group, *n* = 6).

OGD/R-induced Neuro-2a cell injury acts as a model to mimic cerebral I/R injury *in vitro* and results in neuronal insult [18,19]. This model could mimic extracellular condition in cerebral ischemia and subsequently reperfusion, which is used as a common model for research *in vitro*. First, we assessed Neuro-2a cell survival rate after OGD/R. The cell death assay showed 20.98% of the cells died after a 4 h OGD and reperfusion for 6 h. This cell death percentage increased with reperfusion time to 40.25% after 12 h and then decreased to 25.33% after 24 h (Figure 1A), which indicated the most serious injury after OGD/R occurred at 12 h, and then cells recovered and proliferated.

In ischemic cerebral model, autophagy is activated, followed by the rapid elimination of autophagic flux [20]. However, little is known regarding autophagy in the reperfusion phase after cerebral ischemia. OGD/R-induced changes in DRAM1 and certain autophagic pathway proteins in Neuro-2a cells were determined from 6 to 24 h after OGD/R treatment. The ratio of LC3II/I significantly increased and peaked at 12 h after reperfusion (Figure 1B,C), which suggested the presence of autophagy during reperfusion [21]. OGD/R treatment induced a significant increase in DRAM1 protein levels, with a peak at 12 h after reperfusion (Figure 1B,C). Lysosomal-associated membrane protein 2 (LAMP2), a single-span lysosomal membrane protein, which functions as a lysosomal membrane receptor of autophagy, was also investigated [22]. It has been reported that LAMP2 dysfunction results in inefficient autophagosome clearance [23]. OGD/R increased LAMP2 protein levels compared with the control group (Figure 1B,C). Ubiquitin-binding protein (p62), a substrate of autophagy, binds to LC3 and decreases during autophagy [24,25]. We found that p62 levels decreased in OGD/R-induced Neuro-2a cell injury. These data suggested that autophagy was activated in Neuro-2a cells after OGD/R treatment.

### 2.2. Inhibition of Autophagy Exacerbates OGD/R-Induced Neuro-2a Cell Injury

**Figure 2:**
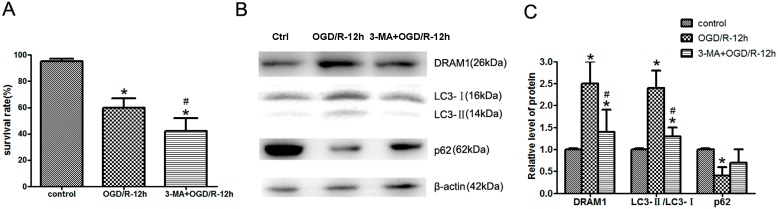
Inhibition of autophagy exacerbates OGD/R-induced Neuro-2a cell injury. Neuro-2a cells were pretreated with the autophagy-specific inhibitor 3-Methyladenine (3-MA) and then subjected to 4 h OGD and 12 h reperfusion. (**A**) Cell viability was assessed using a LIVE/DEAD viability/cytotoxicity kit; (**B**) Relative protein expression levels. At the OGD/R 12 h time point, Neuro-2a cells were collected, and protein was extracted to assess DRAM1, LC3, and p62 expression levels through Western blot analysis. β-Actin was used as an internal control; and (**C**) The quantitative results from (**B**). β-Actin was used as an internal control (* *p* < 0.05 *vs.* control, # *p* < 0.05 *vs.* ODG/R 12 h group, *n* = 6).

To identify the role of autophagy activation in OGD/R-induced Neuro-2a cell injury, we used the autophagy-specific inhibitor 3-Methyladenine (3-MA) to suppress autophagy activation. We found that the increases in DRAM1 expression and the LC3II/I ratio in the Neuro-2a cells treated with OGD/R were significantly inhibited after co-treatment with 3-MA, and the p62 decline was also attenuated (Figure 2B,C). In addition, the survival rate of Neuro-2a cells was markedly reduced after 3-MA treatment with OGD/R (Figure 2A), which contrasts with the ischemic cerebral model [26], indicating that autophagy inhibition exacerbated OGD/R-induced Neuro-2a cell injury and that autophagy may play a protective role in OGD/R-mediated Neuro-2a cell injury [14].

### 2.3. DRAM1 Knockdown Inhibits Autophagy Activation and Aggravates OGD/R-Induced Neuro-2a Cell Injury

**Figure 3:**
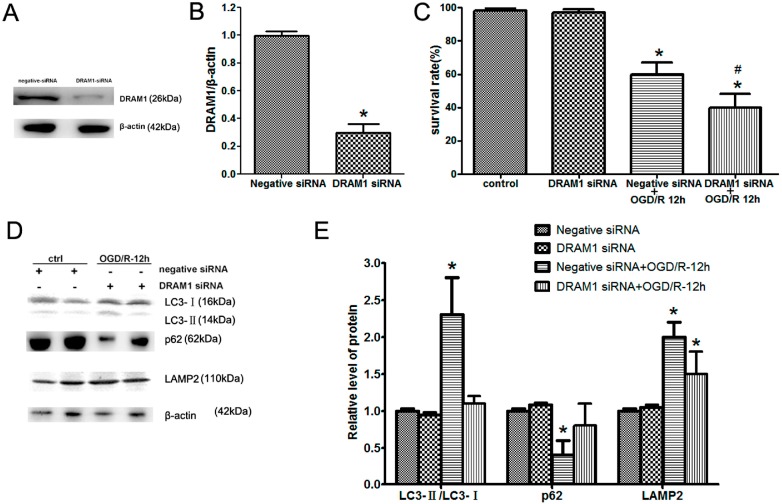
DRAM1 knockdown inhibits autophagy activation and aggravates OGD/R-induced Neuro-2a cell injury. Neuro-2a cells were transfected with negative siRNA or DRAM1 siRNA and then subjected to 4 h OGD and 12 h reperfusion. (**A**) Neuro-2a cells were transfected with negative siRNA or DRAM1 siRNA; (**B**) The quantitative results from (**A**); (**C**) Cell viability was assessed using the LIVE/DEAD viability/cytotoxicity kit; (**D**) Relative protein expression levels. At the indicated pretreatment, Neuro-2a cells were collected, and protein was extracted to assess LC3, p62, and LAMP2 expression through Western blot analysis. β-Actin was used as an internal control; and (**E**) The quantitative results from (**D**). β-Actin was used as an internal control (* *p* < 0.05 *vs.* control, # *p* < 0.05 *vs.* ODG/R 12 h group, *n* = 6).

DRAM1 expression was significantly up-regulated in OGD/R-induced Neuro-2a cells, peaking at 12 h of reperfusion along with the LC3II/I ratio. Meanwhile, 3-MA treatment reduced DRAM1 expression and LC3II/I ratio. These data suggest that the DRAM1 expression pattern and autophagy were regulated in the in the *in vitro* model. DRAM1 encodes a 238-amino acid protein and is highly conserved in a number of species, including humans, mice, zebra fish, *Drosophila*, and *Caenorhabditis elegans* (*C. elegans*) [27,28]. A previous report showed that DRAM1 has a potential tumor-suppressive function and is required for p53-induced autophagy [29]. Although we have demonstrated a protective role of autophagy and increased DRAM1 expression during OGD/R-induced Neuro-2a cell injury, it is unknown how autophagy and DRAM1 protect against cell injury and death. To confirm the links among DRAM1 expression, autophagy activation, and OGD/R-induced Neuro-2a cell injury, we used a siRNA specifically targeting DRAM1. We found that DRAM1 expression in Neuro-2a cells was significantly inhibited after DRAM1 siRNA treatment (Figure 3A,B). The inhibition of DRAM protein production had no effect on the LC3II/I ratio or the levels of p62 and LAMP2 of Neuro-2a cells under normal conditions and resulted in a significant decrease in the LC3II/I ratio and an increase of p62 in OGD/R-induced Neuro-2a cells (Figure 3D,E). Galavotti *et al.* also documented that DRAM1 knockdown inhibits p62 targeting to autophagosomes and reduces its degradation [30]. However, DRAM1 silencing had little effect on LAMP2 expression in OGD/R-induced Neuro-2a cells. LAMP2 is a single-span lysosomal membrane protein [31], and knockout of the entire gene results in abnormal lysosomal biogenesis and inefficient autophagosome clearance [32]. We hypothesized that LAMP2 might show a compensatory increase after DRAM1 knockdown. Given that DRAM1 and LAMP2 are both lysosomal membrane proteins, they likely exert a similar effect on the autophagy-lysosome pathway. Furthermore, the survival rate of Neuro-2a cells was markedly reduced after OGD/R treatment in the absence of DRAM1 (Figure 3C). Our results indicated that siRNAs specific for DRAM1 inhibited autophagy activation and aggravated OGD/R-induced Neuro-2a cell injury. However, there was little effect on LAMP2 expression after DRAM1 knockdown.

### 2.4. DRAM1 Knockdown Blocks Autophagy by Decreasing Autophagosome-Lysosome Fusion

**Figure 4:**
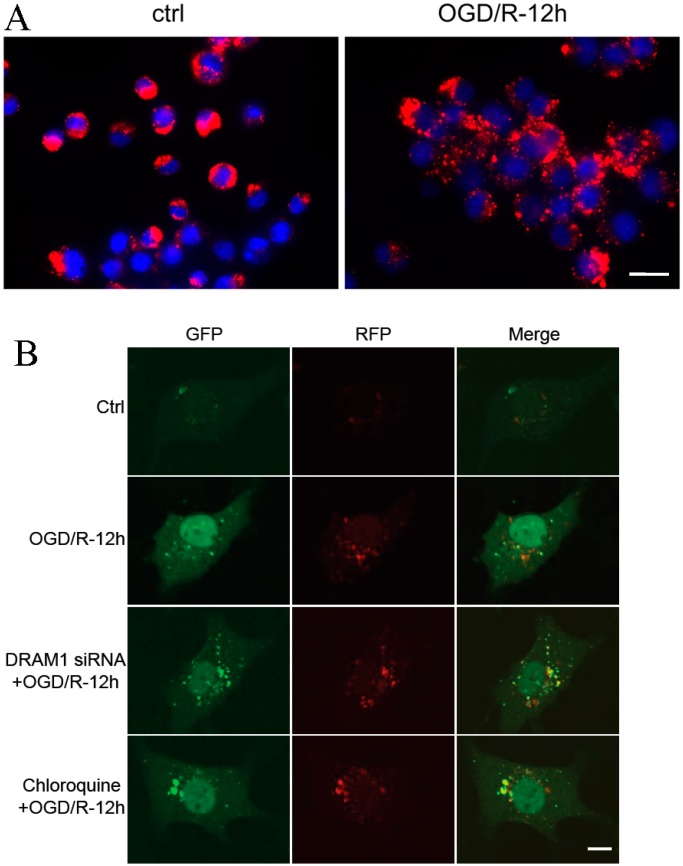
DRAM1 knockdown blocks autophagy by decreasing autophagosome-lysosome infusion. (**A**) After OGD/R 12 h treatment, Neuro-2a cells were incubated with LysoTracker Red (50 mM) for 30 min; and (**B**) After DRAM1 siRNA or chloroquine pretreatment and red fluorescent protein–Green fluorescent protein–microtubule associated protein 1 light chain 3 (RFP–GFP–LC3) reporter plasmid transfection, Neuro-2a cells were subjected to OGD/R 12 h and then observed with confocal microscope. Scale bar = 15 µm.

To investigate the mechanism by which DRAM1 regulates autophagy, we investigated the effects of DRAM1 on autophagosome-lysosome fusion because of its subcellular location. Lysosomes are rich in hydrolytic enzymes and are responsible for the degradation of intracellular materials captured by autophagy. LysoTracker Red was used to stain lysosomes. OGD/R treatment increased the number of LysoTracker-labeled lysosomes (Figure 4A). Neuro-2a cells were transfected with a red fluorescent protein–Green fluorescent protein–microtubule associated protein 1 light chain 3 (RFP–GFP–LC3) expression vector to monitor autophagy [33]. Increases in both autophagosomes and autolysosomes (as indicated by RFP–GFP–LC3) [34] were considered an “intact” flux *in vivo*. After autophagosome formation, autophagosome turnover is mostly determined by the process of autophagosome-lysosome fusion and the degradation of autophagy contents by lysosomal enzymes [35]. Due to differences in acid tolerance, dual fluorescent protein expression vectors were used to observe the autophagy process. When autophagosomes fuse with lysosomes, GFP disappears within the lysosomal environment and RFP remains. Thus, different fluorescence signals may indicate different steps in autophagy. At 12 h after OGD/R, the red LC3 puncta had increased compared with the control group; however, the yellow LC3 puncta were less because the autophagosome clearance was smooth. DRAM1 knockdown increased the yellow puncta (Figure 4B), which suggest that the accumulated autophagosomes in OGD/R-induced Neuro-2a cells are not attributable to inhibited autophagic flux [9]. Pretreatment with chloroquine resulted in a phenomenon similar to DRAM1 knockdown. Chloroquine raises the lysosomal pH and inhibits the fusion of autophagosomes and lysosomes, thus preventing the maturation of autophagosomes into autolysosomes and blocking a late step of autophagy [36]. Lysosome destabilization is induced by DRAM1 in virus-cell interactions, and knocking down DRAM expression with specific siRNAs inhibits autophagy and lysosomal membrane permeabilization [37]. Zhang *et al.* reported that DRAM1 regulates autophagy in a 3-Nitropropionic Acid (3-NP) treatment A549 cell model through promoting lysosomal acidification and activating lysosomal enzymes [38]. Lysosomal pH and permeability might be involved in DRAM1-mediated autophagy. Furthermore, some reports have documented that mitochondria are also involved in DRAM1-mediated autophagy. A mitochondrial inhibitor upregulates DRAM1 expression, and mitochondrial protein synthesis inhibition induces autophagy through the DRAM1 pathway [39]. Autophagy, at least partially, contributes to the neurodegeneration induced by mitochondria dysfunction [40]. DRAM1-mediated autophagy may be a complex process regulated by multi-organelles. These results suggested that DRAM1 knockdown might block autophagy through decreasing autophagosome-lysosome fusion, and the specific mechanism of DRAM1 on autophagosome-lysosome fusion requires further study in the future to fully understand the role of DRAM1 in autophagy.

## 3. Experimental Section

### 3.1. Cell Culture

The mouse neuroblastoma cell line Neuro-2a was purchased from the American Type Culture Collection (ATCC, Manassas, VA, USA) and cultured in Dulbecco’s Modified Eagle’s Medium (DMEM; Gibco, Grand Island, NY, USA) with 10% fetal bovine serum (FBS, Gibco, Grand Island, NY, USA), 100 μg/mL streptomycin (Gibco, Grand Island, NY, USA) and 100 U/mL penicillin (Gibco, Grand Island, NY, USA). The cells were maintained at 37 °C in a saturated-humidity atmosphere containing 95% air/5% CO_2_.

### 3.2. Transfection with the Red Fluorescent Protein–Green Fluorescent Protein–Microtubule Associated Protein 1 Light Chain 3 (RFP–GFP–LC3) Expression Vector and DRAM1 Knockdown

Neuro-2a cells were transfected with the GFP–RFP–LC3 expression vector or DRAM1 siRNA using Lipofectamine 2000 (Invitrogen, Waltham, MA, USA) according to the manufacturer’s instructions. Empty vector controls or negative siRNA controls were used for each transfection. The GFP–RFP–LC3 reporter plasmid (ptfLC3; Addgene, Cambridge, MA, USA) has previously been described [34]. Small interfering RNAs (siRNA) targeting DRAM1 mRNA (5'-CCACGATGTATACAAGATA-3', Invitrogen, Waltham, MA, USA) and a negative siRNA (5'-TAAGGCTATGAAGAGATAC-3', Invitrogen, Waltham, MA, USA) have been described and validated previously [16,41]. The cells were subjected to treatment 48 h after transfection.

### 3.3. Oxygen-Glucose Deprivation (OGD) and Reperfusion

The OGD and reperfusion model was established as described previously [42]. DMEM medium was removed, and the Neuro-2a cells were washed twice with oxygen and glucose-free Hank’s Balanced Salt Solution (HBSS, pH 7.4, Gibco, Grand Island, NY, USA). Then, the medium was replaced with oxygen and glucose-free HBSS, and the cells were transferred to an anaerobic chamber containing 95% N_2_ and 5% CO_2_ at 37 °C. After 4 h, the cells were maintained in DMEM with 10% FBS and returned to normal conditions for 0, 6, 12 or 24 h.

### 3.4. Cell Viability Assay

The cell viability assay was performed using the LIVE/DEAD viability/cytotoxicity kit for mammalian cells (Invitrogen, Waltham, MA, USA). Neuro-2a cells were cultured in 96-well plates for 24–48 h until acceptable cell density levels were reached. The cells were rinsed twice with D-PBS (Gibco, Grand Island, NY, USA) to remove serum esterases and then incubated for 30 min at room temperature in a mixture of 2 μM calcein AM (Invitrogen, Waltham, MA, USA) and 4 μM ethidium homodimer-1 (Invitrogen, Waltham, MA, USA). The live (calcein AM-labeled, green) and dead (ethidium homodimer-1-labeled, red) cells were visualized on a fluorescence microscope (Olympus IX 71, Tokyo, Japan) and automatically counted using ImageJ software (National Institutes of Health, Maryland, MD, USA).

### 3.5. Lysosome Staining and GFP–RFP–Fluorescence Observation

The LysoTracker Red and Hoechst stains were diluted in DMEM. The cells were cultured in 37 °C medium containing 50 nM LysoTracker Red (Sigma-Aldrich, St. Louis, MO, USA) and 10 μg/mL Hoechst (Sigma-Aldrich, St. Louis, MO, USA) for 30 min. The cells were observed and analyzed using a fluorescence microscope. After GFP–RFP–LC3 reporter plasmid transfection and different treatment, the cells were observed and imaged with a confocal microscope (Leica Camera AG, Solms, Germany), more than 30 cells were calculated.

### 3.6. Western Blot Analyzes

Western blotting was performed as previously described [43]. The cells were rinsed twice with ice-cold PBS (Beyotime, Jiangsu, China) and harvested with RIPA lysis buffer (Beyotime, Jiangsu, China). Total protein was quantified and separated on a 10%–15% SDS/polyacrylamide gel (SDS–PAGE, Beyotime, Jiangsu, China) and transferred to nitrocellulose membranes (Millipore, Massachusetts, MA, USA). The membranes were blocked in TBS–Tween buffer containing 20 mM Tris-HCl (Beyotime, Jiangsu, China, 150 mM NaCl, 5% nonfat milk, and 0.05% Tween-20 (pH 7.5) (Beyotime, Jiangsu, China) for 1 h at room temperature and probed with specific antibodies against human LC3 (1:400; Abcam, Cambridge, UK), DRAM1 (1:1000; Abcam, Cambridge, UK), LAMP2 (1:400; Abcam, Cambridge, UK), p62 (1:500; Abcam, Cambridge, UK), and β-actin (1:500; Santa Cruz Biotechnology, Santa Cruz, CA, USA) at 4 °C overnight. The protein levels were normalized to β-actin. The membranes were rinsed five times (5 min each) with PBS-T (Beyotime, Jiangsu, China) and then incubated with an appropriate secondary antibody (diluted 1:10,000 in PBS, 0.01% Tween-20; Thermo Scientific, Maryland, MA, USA) for 1 h, followed with rinsing five times (5 min each) in PBS-T. Immunoreactivity was detected with enhanced chemoluminescent autoradiography (eECL Western Blot Kit, Cwbiotech, Beijing, China) according to the manufacturer’s instructions. The protein expression levels were quantitatively analyzed using ImageJ software.

### 3.7. Statistical Analyzes

All results were expressed as means ± SDs. The differences between multiple groups were computed using a one-way analysis of variance (ANOVA) followed by Tukey’s test. A value of *p* < 0.05 was considered significant.

## 4. Conclusions

In conclusion, we demonstrated that DRAM1 regulated autophagy in an OGD/R-induced mouse Neuro-2a cell injury model. DRAM1 was initially described as regulating autophagy in cancer; here, we extended its role in the context of cerebral I/R. Our study suggested that DRAM1 expression patterns and autophagic pathways were regulated in OGD/R-induced Neuro-2a cell injury. Inhibition of autophagy exacerbated OGD/R-induced Neuro-2a cell injury, which involved the effects of DRAM1 and autophagosome-lysosome infusion. These findings may provide experimental and therapeutic options for the treatment of a broad range of pathologic disorders associated with cerebral ischemia reperfusion injury.
